# Supplementary material for: MetaRibo-Seq measures translation in microbiomes
Source: Nat Commun. 2020 Jun 29;11:3268. doi: 10.1038/s41467-020-17081-z (PMC7324362; doi:10.1038/s41467-020-17081-z)
Supplement: Supplementary file 10 — Supplementary Data 7 [file 41467_2020_17081_MOESM10_ESM.zip › File2/Confidence_High_Taxonomy/319382_out.krona.html]

Javascript must be enabled to view this page.

members
magnitude
magnitudeUnassigned
count
unassigned
taxon
rank

319382\_out

2

2
superkingdom
2

1
1239
phylum


SRS971276\_contig\_number\_contig-100\_858.191730
1879010
species
1

phylum
1224
1

1
class
28216

order
32003
1

1
206379
family


SRS1041132\_contig\_number\_contig-100\_880.37305
1
genus
35798
